# Compositional and biochemical activity evaluation of highly polymerized tea pigments in black tea based on natural deep eutectic solvent extraction

**DOI:** 10.1016/j.fochx.2025.102413

**Published:** 2025-03-25

**Authors:** Di Wang, Yang Liu, Bin Zeng, Yuqin Xu, Sheng Cao, Yuanyan Luo, Shuangling Xiao, Jie Teng

**Affiliations:** a Department of Tea Science, College of Agriculture, Jiangxi Agricultural University, Nanchang 330045, China; b Suichuan Tea Research Institute, Ji'an 343009, China

**Keywords:** Natural deep eutectic solvent, Black tea, Extraction, Separation, Highly polymeric tea pigments

## Abstract

Highly polymeric tea pigments (HPTPs) in black tea have not been comprehensively analyzed because of the complex composition of their structural components. 14 natural deep eutectic solvents (NADESs) were evaluated for extracting HPTPs from black tea, and choline chloride-urea (ChCl-UA) was selected as the best candidate. The HPTPs were separated from the NADES using a dialysis membrane. Microstructural analysis was conducted by XRD, SEM, and AFM, combined with thermal property analysis using TG-DSC and Py-GC–MS determination of biochemical components, and structural analysis through UV–visible, FT-IR, NMR, and SERS spectroscopies. The antioxidant activity analyzed using ABTS, DPPH, and FRAP assays. The results revealed that the extract obtained using the ChCl-UA has a higher tea pigment content, greater polymerization degree, and simpler impurities. The extraction of HPTPs using a NADES and the analysis of the chemical composition and structural characteristics of the extract are helpful for understanding the macromolecular pigments in tea.

## Highlights

• Significantly improved tea pigment extraction using choline chloride-urea as NADES.
• Membrane separation of NADES and highly polymerized tea pigments (HPTPs).
• Relatively more effective extraction of HPTPs using NADES than using water.
• Guidelines for extracting HPTPs extraction and structure–activity relationship analysis.

## 1 Introduction

As a fully fermented tea, black tea has attracted widespread attention and is preferred owing to its unique aroma, taste, and soup-like color, and it accounts for 3/4th of the global tea consumption. Fresh tea leaves contain phenolic compounds such as catechins and flavonoids, which can be oxidized via the key process of fermentation under the enzymatic action of polyphenol oxidase and peroxidase to form tea pigments, such as theaflavins (TFs), thearubigins (TRs), and theabrownins (TBs). TFs are simple polymers of catechins, that contain more than twenty compound structures (Teng et al., 2022). As simple polymeric substances, TFs are further transformed into highly polymeric TRs and TBs under enzymatic oxidation and various non-enzymatic processes. TRs are generally considered to be polyhydroxy polymers formed by the oxidative coupling and other reactions of catechins, TFs, amino acids, and other substances (Long et al., 2023). Furthermore, the composition of TBs is relatively more complex because they are formed by the further enzymatic oxidation of the catechins, TFs, and TRs. In fact, TBs are high polymers formed by the complexation of polysaccharides, amino acids, proteins, and other substances (Xu et al., 2023). Owing to their complex structures, TRs and TBs are usually referred to as highly polymeric tea pigments (HPTPs). As a natural pigment, tea pigment has attracted widespread attention from researchers owing to its natural coloring and preventive health care functions, and has been widely applied in food and medicine, health care, and daily chemical products (Zhao et al., 2023). As an important component that determines the color of black tea, the structure, composition, chemical properties, and formation mechanism of HPTPs need to be studied further.

The pigments in black tea are usually extracted using water or an organic solvent under boiling conditions, and based on this extraction technique, auxiliary extraction technologies, such as those using microwave, ultrasonic, pulsed electric field, and supercritical fluid (Raghunath et al., 2023). A natural deep eutectic solvent (NADES), which is a homogeneous solvent composed of a hydrogen-bond acceptor (HBA) and hydrogen-bond donor (HBD) in a specific molar ratio, is a promising solvent for the extraction or separation of natural products. NADESs are gradually replacing water and organic solvents as highly efficient extraction solvents owing to the advantages of their simple syntheses, easy degradation, high extraction efficiency, and eco-friendliness (Florindo et al., 2014). Thus, NADESs have been widely applied in the extraction of anthocyanins, flavonoids, polysaccharides, proteins, natural pigments, and other biomolecules (Guzmán-Lorite et al., 2022; Liu et al., 2022; Wang et al., 2024; Zuo et al., 2023). NADESs have been suggested to exist naturally in organisms as a third liquid phase, and many secondary metabolites may continue to participate in biosynthesis with the aid of a NADES (Choi et al., 2011). Therefore, NADESs have a unique advantage as solvents for the extraction of natural products. However, the vapor pressure of NADESs is low, that is, they are non-volatile, which makes the traditional methods of rotary evaporation and freeze-drying unsuitable for the separation and recovery of the NADES used in extraction. At the same time, the strong hydrogen bonds of NADESs make them more effective as solvents to extract target products in the early stages, and it is more difficult to separate NADESs and the target products in the later stages, which makes the separation and recovery of natural products from NADESs considerably challenging. In the traditional method of tea extraction, TRs are obtained by extracting black tea with ethyl acetate and *n*-butanol (Wang et al., 2018), while TBs are obtained by freeze-drying the substances remaining in the aqueous solution (Zhang, Ying, et al., 2024; Zhang, Yu, et al., 2024). Currently, the direct separation of tea pigments from NADESs using methods such as freeze-drying and organic solvent extraction has limitations. Therefore, the separation of the NADESs from natural products and their recycling remain major challenges. The effective separation of the NADES and tea pigments is thus a key factor for considerably improving the greenness and economic efficiency of using NADESs in tea extraction and for ensuring that tea pigments and other substances are effectively isolated at an industrial scale.

Liquid-liquid extraction is one of the main methods used to separate the natural products from the NADES used for their extraction. This process involves the dilution of the natural product-containing NADES with water and then recycling certain compounds, such as flavonoids using ethyl acetate and *n*-butanol (Xu et al., 2019). Further, the method of phase switching is used to convert a switchable hydrophilic eutectic solvent to a hydrophobic one, such as in the separation of extracted hydrophobic *β*-carotene (Stupar et al., 2021). The solvent precipitation method, in which poor solvents (such as an excess of ethanol) is added to the NADES to reduce the solubility of the extracted products, decreases the solubility of TBs extracted from black tea in the mixed system (Liu et al., 2022). Further, high-speed counter-current chromatography was used to successfully recover the target compounds in chrysanthemum from the NADES extract (Wang et al., 2020). In macroporous resin-adsorption method, natural molecules are adsorbed onto a macroporous resin in a column and then washed with aqueous alcohol solutions of different concentrations (Zhao et al., 2024). Reduced-pressure distillation, liquid–solid extraction, aqueous two-phase extraction, crystallization recovery methods, and other special recovery methods can also facilitate the partial recovery of the NADES (Yu et al., 2024). However, the equipment used in the above separation method is expensive or the operation is complex; therefore, we explored a membrane separation method, in which the components are separated based on their molecular weight. Generally, a NADES, as a small molecule, can be removed using an ordinary semi-permeable membrane with a molecular weight cut-off 500–1000 Da, whereas the remaining macromolecular substances, such as polyphenols, polysaccharides, and lignin, are retained on the membrane. TBs and TRs are highly polymerized polyphenols, and studies have shown that the molecular weight of TRs ranges from 5 to 40 KDa while that of TBs exceeds 25 KDa (Xiao et al., 2020). In this study, we not only explored a method for the efficient extraction of tea pigments using a NADES, but also realized the separation of HPTPs and NADES using a membrane. In addition, we comparatively evaluated the biochemical properties of the HPTPs extracted using the NADES with those of the samples extracted using an aqueous solution (traditional method) to verify the effectiveness and influence of the NADES in the extraction of HPTPs from black tea.

## 2 Materials and methods

### 2.1 Materials

Jiangxi Gougunao black tea, made in May 2023, and ground and crushed after 60 mesh sieve reserve. Dialysis membranes (molecular weight cut-off: 3500 Da) were purchased from Changde BKMAM Biotechnology Co., Ltd. (Changde, Hunan Province, China). All chromatographic-grade chemical standards were purchased from the Shanghai Yuanye Biological Technology Company (Shanghai, China). The purity of the chemical standards was no less than 98 %.

### 2.2 Preparation and physical characteristics of NADESs

ChCl was used as the common HBA, and four types of HBDs were screened: organic acids, alcohols, sugars, and amides (Table 1). The HBA and HBD were mixed according to the required molar ratio, and the mixed was heated with stirring in a 90 °C water bath to form a uniform transparent liquid. Conductivity, viscosity measurements, and Fourier transform infrared (FT-IR) spectral analysis were performed at room temperature.

**Table 1:** The details of NADESs used in this study.

NO.	Abbreviation	HBA	HBDs	HBDs structure	Category	Molar ratio
1	ChCl-AA	Choline chloride	Acetic acid	CH_3_COOH	Organic acids	2:5
2	ChCl-OA		Oxalic acid	H_2_C_2_O_4_		1:1
3	ChCl-PA		Propionic acid	CH_3_CH_2_COOH		2:3
4	ChCl-SA		Succinic acid	C_4_H_6_O_4_		3:2
5	ChCl-MA		Malic acid	C_4_H_6_O_5_		3:2
6	ChCl-CA		Citric acid	C_6_H_8_O_7_		1:2
7	ChCl-EG		Ethylene glycol	(CH_2_OH)_2_	Alcohols	1:2
8	ChCl-GL		Glycerol	C_3_H_8_O_3_		1:2
9	ChCl-BD		1,4-Butanediol	C_4_H_10_O_2_		1:2
10	ChCl-XL		Xylitol	C_5_H_12_O_5_		2:1
11	ChCl-SL		Sorbitol	C_6_H_14_O_6_		5:2
12	ChCl-XE		Xylose	C_5_H_10_O_5_	Sugar	2:1
13	ChCl-GE		Glucose	C_6_H_12_O_6_		2:1
14	ChCl-UA		Urea	CH_4_N_2_O	Amides	1:2

### 2.3 Determination of the tea pigment contents

The reference method was used with appropriate modifications (Cui et al., 2022). The black tea sample (0.4 g) was added to 25 mL of the prepared NADES or water. The mixture was soaked in boiling water for 5 min and then centrifuged at 4500 rpm for 10 min, after which the supernatant was collected. Subsequently, an additional 25 mL of NADES or water was added to the tea residue, and the extraction process was repeated. The two supernatants were combined to obtain 50 mL of the extract. Next, to 2 mL of the black tea extract in the NADES, 2 mL of a saturated oxalic acid solution and 6 mL H_2_O were added, and the volume was adjusted to 25 mL using 95 % ethanol. The absorbance, E_A_ of the tea pigment solution was recorded at 380 nm with the use a 95 % ethanol in water as a control. The content of tea pigment was calculated as follows:

(1) $$X_{\mathrm{TP}}=\left(C\times E_{A}\right)/\left(100\times 0.4\right)$$

where C is the Roberts empirical coefficient (C = 16.944).

### 2.4 Extraction of tea pigments using the NADES

Using the same extraction parameters mentioned above, 14 NADES samples were tested to evaluate their efficiency in extracting tea pigments, leading to the selection of the most suitable NADES candidate. Single-factor experiments were conducted based on the optimal NADES to investigate the effects of the extraction time (4, 7, 10, 13, 16, and 19 min), extraction temperature (50, 60, 70, 80, 90, and 100 °C), NADES water content (0, 10, 20, 30, 40, and 50 %), and solid–liquid ratio (1:50, 1:75, 1:100, 1:125, 1:150, and 1:175 g/mL) on the extraction efficiency of tea pigments. Subsequently, a response surface model was applied using Design-Expert 8.0 software (Stat-Ease Inc., USA) to conduct a three-levels analysis with three independent variables (Table S1). The complete design comprised 17 experimental points, including five replications of the center points, aimed at determining the optimal extraction conditions.

### 2.5 Separation and preparation of HPTPs

**Fig. 1:**
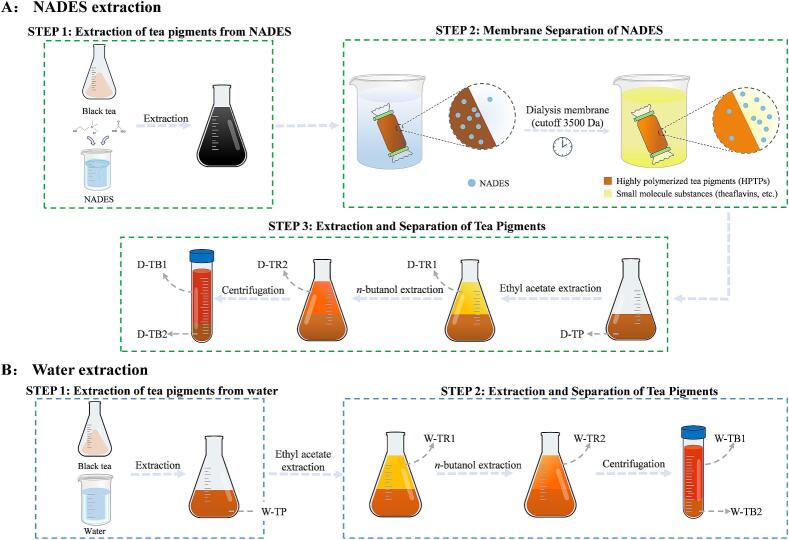
Flow chart of the extraction and separation of tea pigments using a NADES (A). Flow chart for the extraction and separation of tea pigments using pure water (B).

A known volume (25 mL) of the NADES extract of the tea pigments was loaded into a dialysis membrane (cutoff 3500 Da) and heated at a constant temperature of 60 °C under magnetic stirring. During this time, pure water was replaced every 4 h until the color ceased to change. The total tea pigment in the dialysis membrane (D-TP) was collected. The collected aqueous solution was extracted thrice with equal volumes of ethyl acetate to obtain a TR solution denoted as D-TR1. It was then extracted thrice with equal volumes of *n*-butanol to obtain a TR solution denoted as D-TR2. The remaining aqueous solution was centrifuged at 4 °C and 8000 rpm for 10 min, and the bottom residue of TBs was collected to precipitate D-TB2, and finally an aqueous solution of TBs (D-TB1) was obtained. The collected solutions were subjected to rotary evaporation and then freeze-dried to obtain powders (Fig. 1). The method for isolating tea pigments from water is same as that for the NADES case. The quantity of each portion of the collected tea pigment was determined by weighing, and the percentage of a certain tea pigment in the total amount of the tea pigment was calculated.

### 2.6 Morphological characteristics of HPTPs

#### 2.6.1 Scanning electron microscopy (SEM)

The morphological characteristics of the HPTPs were analyzed using a scanning electron microscope (SEM; Regulus 8100, Hitachi, Japan). The powder sample was sputtered with gold under vacuum, and SEM images were captured.

#### 2.6.2 Atomic force microscopy (AFM)

The surface morphology, nanostructure, and chain conformation of the HPTPs were studied using an atomic force microscopy (AFM; Bruker, Karlsruhe, Germany). The nanoparticle size and surface roughness of the materials were analyzed using AFM images. For AFM analysis, the HPTPs were first dissolved in water and then dried to a 5 μL solution (10 μg/mL) on the surface of mica.

### 2.7 Determination of the chemical components of the HPTPs

#### 2.7.1 Determination of the main biochemical components

The total amount of tea polyphenols was determined using the folin–phenol method, and the total amount of free amino acids was determined using ninhydrin hydrate colorimetry (Chen, Fu, et al., 2024; Chen, Wang, et al., 2024). The total polysaccharide content was determined using the phenol–sulfuric acid method. The total flavonoids content was determined using the aluminum nitrate colorimetric method. Caffeine was quantified using high-performance liquid chromatography (HPLC; Agilent, CA, USA) (Hua et al., 2021). The protein content was determined using a Micro BCA Protein Assay Kit (Jiancheng Bioengineering Institute, Nanjing, China), according to manufacturer's instructions, and bovine serum albumin was used to draw a standard curve (Xu et al., 2024).

#### 2.7.2 Pyrolyzer-gas chromatography-mass spectrometry (Py-GC–MS)

The pigment powder was pyrolyzed at 280 °C for 5 s using a CDS 5000 pyrolyzer. Then, the GC–MS analysis of the pyrolysis products was performed on GC/MSD equipment (Agilent, CA, USA) using a DB-5 MS column. The column temperature was first held at 40 °C for 1 min, then increased to 200 °C at 8 °C/min and held there 2 min, and then increased to 280 °C at 10 °C/min and held there for 5 min. Helium (99.999 %) used as the carrier gas was flown at 1 mL/min rate, the mass range was 10–800 *m*/*z*, and the electron ionization energy level was 70 eV. The interface temperature was 280 °C, and the ionization source temperature was 230 °C. The compounds were retrieved using NIST14 and quantified through peak area normalization.

#### 2.7.3 Thermogravimetry (TG)-differential scanning calorimetry (DSC)

The tea pigment samples were accurately weighed in an alumina crucible and gradually heated from 30 to 1200 °C at 10 °C/min for TG-DSC analyses (TGA/DSC3+, Mettler Toledo, Switzerland). Nitrogen (99.999 %) was used as the purification gas, and the flow rate was set at 20 mL/min. Derivative thermogravimetry (DTG) data were obtained by differentiating the thermogravimetry data.

#### 2.7.4 X-ray diffractometry (XRD)

XRD analysis (SmartLab SE, Rigaku, Japan) of the tea pigments was conducted in the diffraction angle (*2θ*) range of 10 to 80° at a scan speed of 1°/min. The X-ray generator was operated at 3 kW, and the goniometer radius was 300 mm.

### 2.8 Spectral analysis of the HPTPs

#### 2.8.1 UV–visible spectroscopy

The tea pigment powders were weighed and dissolved in pure water to prepare 0.2 mg/mL solutions for UV–visible spectral analyses (UV-2600, Shimadzu, Japan). The solution was taken in a 10 mm quartz cuvette and scanned at a slit width of 1 nm. The absorbance spectrum of the solution was recorded in the 200–600 nm range.

#### 2.8.2 FT-IR spectroscopy

FT-IR spectroscopy (Nicolet iS50, Thermo Fisher Scientific, USA) was performed using the KBr method. The tea pigment powder was mixed with KBr to form a blended powder that was pressed into a pellet. The FT-IR spectra were obtained at a resolution of 8 cm^−1^ in the wavenumber range of 400–4000 cm^−1^; spectral scans were repeated 32 times.

#### 2.8.3 Surface-enhanced Raman scattering (SERS)

The tea pigment samples were weighed and dissolved in pure water to prepare 1 mg/mL solutions. SERS measurements (RM2000, Renishaw, UK) were performed using nano‑gold for signal enhancement. First, 300 μL of the prepared nano‑gold gel and 100 μL of a 1.5 % sodium chloride solution were added to a quartz injection bottle. Next, 40 μL of the tea pigment solution to be analyzed was added. Raman spectra were collected in the 400–2000 cm^−1^ range at 1 cm^−1^ resolution. The acquisition parameters were as follows: excitation wavelength, 785 nm; laser power, 400 mW; integration time, 10 s; number of spectral integrations, 2. The asymmetric least-squares smoothing baseline method was used to subtract the baseline from the data for peak analysis.

#### 2.8.4 Nuclear magnetic resonance spectroscopy (NMR)

The tea pigment powder was dissolved in D_2_O, and the ^1^H and ^13^C NMR spectra of the sample were acquired using an NMR spectrometer (Bruker 400 M, Bruker, Germany) with a working frequency of 600.35 MHz at room temperature. Tetramethylsilane was used as the internal reference (8H0.00). MestReNove 14.2 software (Mestrelab Research, Santiago, Spain) was used to process all raw NMR spectral data.

### 2.9 Determination of the antioxidant activity of the HPTPs

The antioxidant activity of the extracted tea pigments was determined by evaluating their reaction with DPPH^•^, ABTS^+^, and FRAP. Tea pigment solution were prepared at different concentrations and analyzed using relevant kits (No.: DPPHFRS-F48S-N1620), TAOCA-F48S-N (1620), and TAOCA-W96S-N (1620), Enzyme-linked Biotechnology Co., Ltd., Shanghai, China). Ascorbic acid was used as a positive control.

### 2.10 Data analysis

Data are expressed as mean ± standard deviation. Graphs were produced using GraphPad Prism 8. Statistical analysis was performed using SPSS software. *P* < 0.05 was considered statistically significant.

## 3 Results and discussion

### 3.1 Characteristics of NADES samples

The structural changes in the NADES samples were analyzed using FT-IR and SERS techniques. The results revealed that the NADES exhibit the characteristic peaks of the constituent HBA and HBD. The FT-IR spectra of ChCl-acids were used as examples (Fig. S1), the organic acid retained the absorption peak of 1710 cm^−1^ for the –COOH group in HBD. In addition, the molecular vibrations of the O—H bond appeared at 3400 cm^−1^, and the absorption peak intensity of the NADES in this region was different from those of the corresponding HBA and HBD, indicating the synthetic characteristics of the NADES and the influence of hydrogen bonding on the molecular structure. The SERS spectrum in Fig. S2 shows that ChCl used as the HBA presents an absorption peak at approximately 710 cm^−1^, and the synthesized NADES exhibited the same absorption peak in this region. In addition, the NADES prepared using other HBAs and HBDs showed absorption peaks at 490 and 1320 cm^−1^. The FT-IR and SERS results confirmed that the NADES was formed by the hydrogen bonding of the HBA and HBD (Xu et al., 2024).

The conductivity and viscosity of a NADES affect the extraction of natural products. Therefore, the conductivity of the NADES was evaluated (Fig. S3); NADES derived from organic acids and amides showed higher conductivities, whereas those derived from polyols and sugars showed lower conductivities. The viscosities of the NADES are shown in Fig. S3. The viscosities of the ChCl-SA, ChCl-MA, ChCl-CA, and sugar NADES were higher than those of the other NADES. The low viscosity of some NADES may be related to the structure of the corresponding HBD. A NADES synthesized using an HBD with a relatively simple structure and fewer carbon atoms has a lower viscosity (Abbott et al., 2003; Zuo et al., 2023). These results confirmed that the 14 NADESs selected in the experiment were successfully prepared.

### 3.2 Extraction of tea pigments using different NADESs

**Fig. 2:**
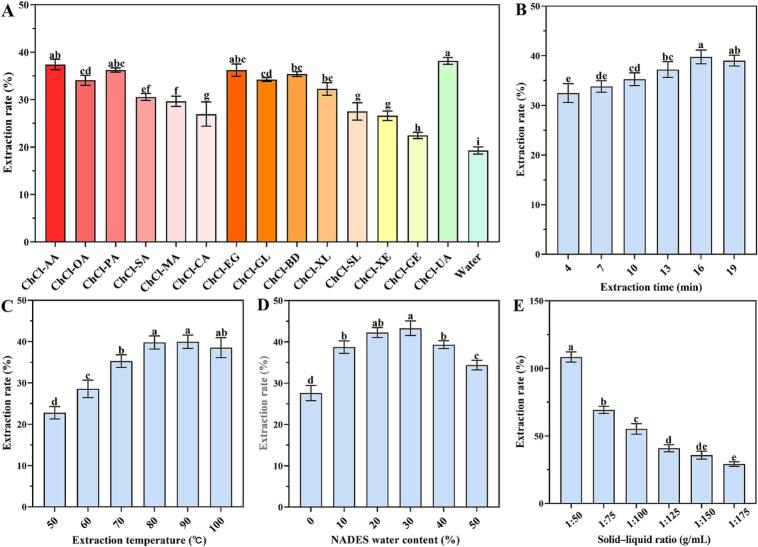
Effects of different NADES samples on the extraction rate of tea pigments (A). Effects of the extraction time (B), extraction temperature (C), NADES water content (D), and extraction solid–liquid ratio (E) on the extraction rate of the tea pigments. The statistical differences between the data are represented using different letters (*P* < 0.05).

The results indicated that NADESs are suitable for extracting black tea pigments, and the tea-pigment extraction efficiencies of the 14 NADESs were better than that of water-based extraction (Fig. 2A). ChCl-UA provided a significantly higher tea-pigment extraction efficiency (38.16 %) than the other NADESs; this value is almost twice that of water extraction (19.28 %). In addition, ChCl-EG with a polyol HBD had an excellent tea-pigment extraction effect, while the extraction effect of ChCl-AA with an organic acids as the HBD was second only to that of ChCl-UA. The tea-pigment extraction efficiency of the sugar-based NADES was lower than those of the other NADESs. Urea, acetic acid, and ethylene glycol have simple structures, and the NADESs of the same mass synthesized using these HBDs have more hydrogen bonds, which may explain their good effect on the extraction of tea pigments (Guzmán-Lorite et al., 2022). Overall, ChCl-UA exhibited the highest extraction efficiency, surpassing that of the optimal NADES reported for the extraction of TRs and TBs in other studies (Liu et al., 2022; Zhao et al., 2024). The analysis indicated that the simple structure of ChCl-UA facilitates its binding to tea pigments. Furthermore, the -NH^2^ groups in urea and the -OH group in ChCl form stronger hydrogen bonds with the natural products, which effectively enhances their binding with a greater quantity of tea pigments. Additionally, ChCl-UA disrupts the structure of plant cellulose and hemicellulose (Zhang, Ying, et al., 2024; Zhang, Yu, et al., 2024), aiding the release and binding of tea pigments. Therefore, ChCl-UA was selected as the optimal candidate to extract the tea pigments from black tea.

### 3.3 Single factor test results

An adequate extraction time facilitates the interaction of the NADES with the natural products (Fig. 2B). Prolonging the extraction time is beneficial for the precipitation and dissolution of tea pigments from black tea, and it allows the NADES to extract more tea pigments. Upon heating for 16 min, the tea-pigment extraction rate of the NADES did not decrease significantly. As the temperature increases, the viscosity, surface tension, and density of the NADES decrease, and the diffusion coefficient increases, which can promote the dissolution of biomolecules, such as tea pigments, leading a higher extraction rate (Fig. 2C). When the temperature is increased in the range of 50–100 °C, solvent adsorption is enhanced, which promotes the dissolution of the pigments in tea. At 80–100 °C, the extraction rate of the tea pigments stabilized over a certain range, it did not decrease significantly. Catechins have high thermal stability, and being catechin polyphenol polymers, tea pigments showed better thermal stability.

The tea-pigment extraction rate increased with increasing water content in ChCl-UA and reached the maximum when the water content reached approximately 30 %; thereafter, it gradually decreased with a further increase in the water content in ChCl-UA (Fig. 2D). A higher water content reduces the viscosity and surface tension of ChCl-UA, which is beneficial for the dissolution of tea pigments. However, a high water content can also damage the structure of ChCl-UA, resulting in higher diffusion rates between urea and anions, and weakened hydrogen-bonding interactions (Shah & Mjalli, 2014). An appropriate solid–liquid ratio can reduce solvent waste and increase the yield of the extracted product. The results revealed that the extraction rate of tea pigments increased as the tea-to-solvent ratio increased (Fig. 2E). Thus, increasing the solid–liquid ratio to an optimal level can significantly improve the extraction efficiency of ChCl-UA.

### 3.4 Response surface analysis and validation of the regression model

As the solid–liquid ratio increased, the ability of ChCI-UA to extract tea pigments exhibited an upward trend, with the pigment content surpassing the optimal detection range of the analytical method employed. Notably, at a solid–liquid ratio of 1:50, the tea-pigment extraction rate exceeded 100 %; this is, primarily due to the limitations of the detection method. Concurrently, the extraction rate of the tea pigments continued to decline with increasing solid-to-liquid volume ratio, without reaching a peak value. This observation further indicated that the solid–liquid ratio is not appropriate for establishing of a response surface model. Consequently, we adopted the standard solid–liquid ratio of 1:125 g/mL, defined by the tea-pigment detection method, as a specific single factor parameter to establish the response surface experiments focusing on the extraction time, extraction temperature, and the content of ChCl-UA in the extraction of tea pigments using this NADES (Table S1 and Table S2).

**Fig. 3:**
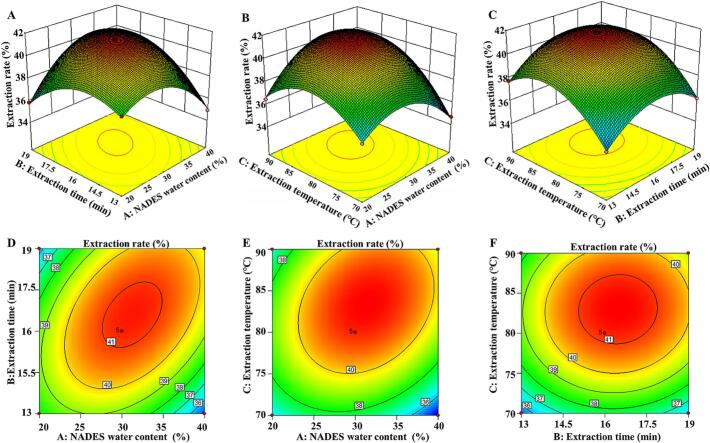
Interaction response surface diagram and contour plot of each factor corresponding to the extraction of black tea pigments using a NADES.

The contour shape of the response surface indicates the interaction between two interaction factors. According to the analysis (Fig. 3), the contours of the water content and extraction time, as well as those of water content and extraction temperature are elliptical, indicating that the water content of ChCl-UA has a significant effect on the extraction of tea pigments. The contour between the extraction temperature and time is circular, indicating that the interaction between these two is not significant. Under the same conditions, a NADES with an appropriate moisture content has the most significant effect on the extraction rate of tea pigments. The regression model for the tea-pigment extraction rate was obtained through a Box–Behnken response surface optimization experiment (Table S3), and the corresponding equation is.

(2) $$Y\;\left(\%\right)=41.18+0.22\;A+0.53\;B+1.30C+1.69\;\mathrm{AB}+1.14\;\mathrm{AC}+0.19\;\mathrm{BCE}-2.24\;A^{2}-1.86\;B^{2}-2.22\;C^{2}$$

The optimal process parameters for tea-pigment extraction using ChCl-UA as the solvent were determined to be as follows: NADES water content of 29.8 %, extraction time of 17.9 min, and extraction temperature of 85.8 °C; the corresponding extraction efficiency of tea pigments is 40.56 %. Furthermore, for practical operation, the optimized NADES water content is 30 %, the extraction time is 18 min, and the extraction temperature is 85 °C; the corresponding average extraction efficiency of tea pigments is 40.3 ± 1.3 %, which is close to the theoretical value (*P* > 0.05), indicating that the process technology for the optimal extraction of tea pigments based on the Box–Behnken response surface is reliable.

### 3.5 Effect of the NADES on the structure of black tea pigments and the HPTPs extraction efficiency

**Fig. 4:**
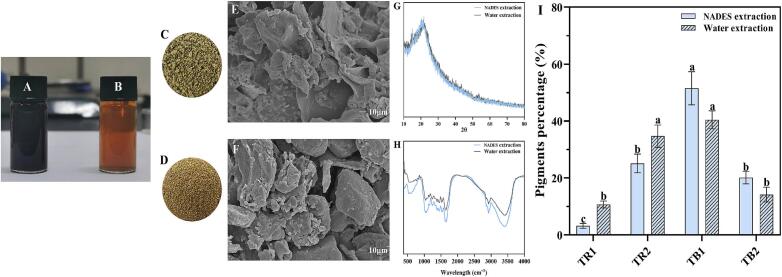
Tea pigment solutions obtained after extraction using ChCl-UA (A) and water (B). Photographs of the black tea residues extracted using ChCl-UA (C) and water (D) as solvents. SEM images of the black tea residues extracted using ChCl-UA (E) and water (F). XRD patterns (G) and FT-IR spectra (H) of the black tea residues obtained using ChCl-UA and water as extraction solvents. Mass percentages of thearubigins and theabrownins separated and dried from the black tea extracts of ChCl-UA and water (I). The statistical differences between the data are represented using different letters (*P* < 0.05).

A color comparison of the tea residue obtained using ChCl-UA as the extraction solvent with that extracted using water (Fig. 4) revealed that the black tea residue obtained after ChCl-UA extraction was yellowish green, while that obtained after water extraction was brownish red (Fig. 4A-D). SEM observations (Fig. 4E and Fig. 4F) revealed that the surface of the tea residue extracted with ChCl-UA was rougher and mostly flaky, whereas the surface of the tea residue extracted with water was smoother and mostly blocky. These results indicate that ChCl-UA destroyed the structure of black tea more significantly, leading to great more tea-pigment dissolution. The XRD patterns (Fig. 4G) and FT-IR spectra (Fig. 4H) of the black tea powders obtained via NADES and water extraction showed similar characteristics, indicating that the tea pigment extracted using ChCl-UA improved the dissolution of the tea pigment in the black tea powder through hydrogen bonding and other intermolecular forces and that the chemical structure of the black tea components did not change.

Through membrane separation, the color of the tea pigment solution in ChCl-UA was changed from uniform black to turbid brown, and the extraction effect of the organic solvent on the solution was significantly improved. The TRs and TBs in the NADES extract were collected, and the percentages of different pigments, viz., TR1, TR2, TB1, and TB2 in the total mass of collected pigments were calculated (Fig. 4I). The proportions of tea pigments obtained using both methods (NADES and water extraction) were in the order of TB1 > TB2 > TR2 > TR1. The percentages of TB1 (51.52 %) and TB2 (20.12 %) obtained using ChCl-UA were significantly higher than those obtained using water (TB1: 40.43 % and TB2: 14.17 %). However, TR1 obtained via NADES extraction (3.23 %) was lower than that obtained via water extraction (10.7 %). During membrane separation, many small molecular substances are removed; consequently, HPTPs were mainly obtained from the ChCl-UA extract.

### 3.6 Analysis of the morphological characteristics of the HPTPs

**Fig. 5:**
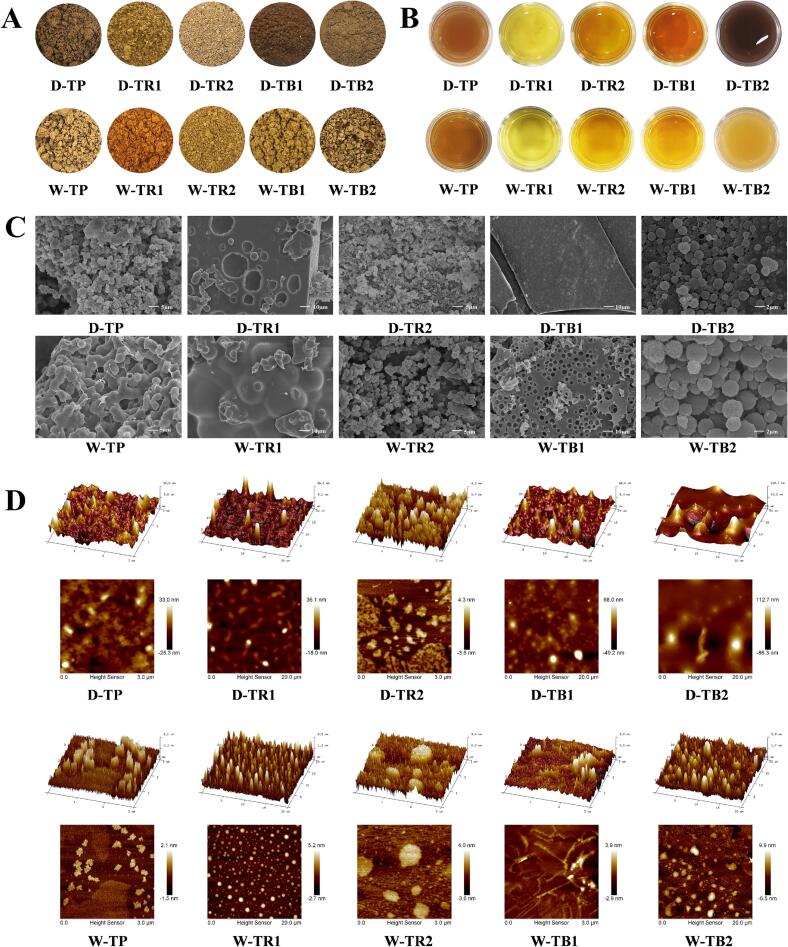
NADES and water extraction of the tea pigment powder (A), photographs of the tea pigment solutions (B), SEM images (C), and AFM (D) images.

As shown in Fig. 5, the tea pigment powder obtained via NADES extraction was overall brown, while the tea pigment powder obtained via water extraction appeared yellowish brown. Small-molecular pigments are retained during water extraction, resulting in the brownish red color of tea pigments such as W-TR1. In the separation of TB2, it was found that TB2 was insoluble in water and caused the solution to become turbid; however, the solution regained its clear and bright color after centrifugation and precipitation. According to colorimetric analyses (Table S4), TR1 had the highest brightness, TR2 had a yellow hue, and TB1 had the reddest hue, whereas TB2 exhibited turbidity, with its brightness being dulled and red and yellow pigment contents being the lowest. At the same time, the color of the tea pigment obtained via NADES extraction was redder, but the brightness and yellow coloring were lower than those of the water-extracted sample.

Further SEM observations revealed that NADES- and water-extracted tea pigment powders had similar microstructures (Fig. 5C). The SEM images reveal that D-TP and W-TP have a rough surface and are composed of irregularly clumped polymers. TR1 has a smooth surface and is composed of semispherical polymers. In contrast, TR2 has a rougher surface and is composed of irregularly clumped polymers, which are smaller and more clustered. TB1 has a smoother surface and is tightly held together by much smaller materials. TB2 has densely packed spherical structures accumulated on its surface. It is generally suggested that the structure of tea cream substances is formed by the combination of caffeine, catechins, TFs, organic acids, and other protein-based substances (Chen, Fu, et al., 2024; Chen, Wang, et al., 2024). However, subsequent studies have shown that TB2 is a mixture similar to tea cream or tannins. It is speculated that hydrophobic groups, such as nonpolar side chains, in the substance may encapsulate small molecules, whereas polar side chains maintain contact with water on the external surface, forming a spherical structure. This spherical structure is stable, insoluble in water, and can cause the extract to become turbid.

The microstructure and distribution of tea pigments were studied using AFM (Fig. 5D). The nanoparticle size, degree of aggregation, and roughness of the tea pigment powders obtained via NADES extraction were greater than those of the samples extracted using water. The molecular aggregation of the tea pigments extracted from NADES was more pronounced, resulting in larger and rougher clusters. Analyses indicated that the dialysis process removed small molecules, and the hydrogen bonding of ChCl-UA may have caused the hydroxyl groups of the extracted tea pigment molecules to bind tightly, forming large molecular clusters (Xiao et al., 2023). The tea pigment molecules extracted from water were relatively scattered; they were distributed in the form of numerous small clumps relatively smooth surfaces; among the water-based samples, W-TB1 and W-TB2 formed island-like or granular structures. In addition, TR1 and TR2 were uniformly distributed as small molecules surrounding some polymeric molecules, whereas TB1 and TB2 showed a more concentrated and clustered polymer composition; that is, the polymerization degree of TBs was significantly higher than that of the TRs.

### 3.7 Chemical components of the HPTPs

#### 3.7.1 Main biochemical components of the HPTPs

**Fig. 6:**
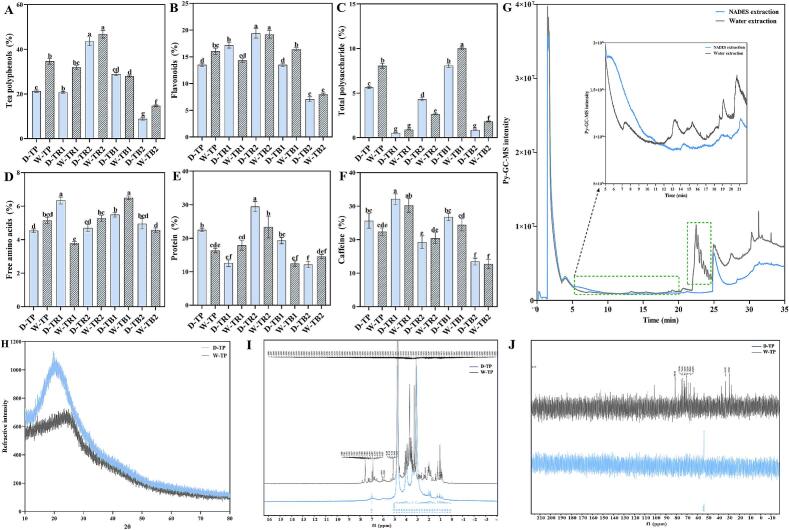
Contents of tea polyphenols (A), flavonoids (B), total polysaccharides (C), free amino acids (D), protein (E), and caffeine (F) in tea pigments extracted using a NADES (ChCl-UA) and water as extraction solvents. Py-GC–MS data (G), XRD patterns (H), ^1^H NMR spectra (I), and ^13^C NMR spectra (*J*) of D-TP and W-TP isolated using ChCl-UA and water as extraction solvents. The statistical differences between the data are represented using different letters (*P* < 0.05).

The chemical components of the tea pigments extracted using the NADES and water as solvents were similar (Fig. 6A-F). The contents of tea polyphenols and flavonoids in TR1 and TR2 were high, but more polysaccharides were retained in the aqueous phase (TB1). Further, the free amino acids and caffeine were evenly distributed in each tea pigment. The protein content in TR2 was relatively high. In addition, the contents of tea polyphenols, flavonoids, total polysaccharides, and proteins in TB2 were low; these are difficult to dissolve in water and can make the tea brew turbid. Tea cream can cause tea brew to become cloudy, and it contains abundant substances and has good antioxidant and anti-inflammatory effects (Qin et al., 2022). We speculate that TB2 has an important effect on the formation of tea cream; however, it is not a traditional substance after cold, and the specific composition of TB2 remains to be investigated further. In conclusion, the content of each component in the tea pigments extracted from ChCl-UA and water was similar.

#### 3.7.2 Py-GC–MS analysis of the HPTPs

The tea pigment components obtained by the two extraction methods were analyzed by Py-GC–MS (Tables S5 and S6). A total of 33 pigment substances were found in the water extract. The presence of phenol (0.883 %), resorcinol (0.385 %), and various heterocyclic compounds indicated that phenolic compounds are important components of tea pigments, and nitrogen-containing compounds may be the decomposition products of proteins and amino acids (Li et al., 2023). With the increase in temperature, various heterocyclic compounds appeared, such as 4-acetylphenylether (8.28 %), 9H-thioxane-9-one, 2-(1-methylethyl)- (8.839 %), hexadeca-2,6,10,14-tetraen-1-ol,3,7,11,16-tetramethyl-(12.661 %), and 2-azetidinone,1-(diphenylacetyl)-3, 3,4-triphenyl- (7.958 %). These substances have complex structures and were found in large proportions, which may be due to the cleavage of the high polymers in tea pigments at high temperatures.

The tea pigments extracted from ChCl-UA were primarily composed of ketene (41.479 %) and caffeine (48.871 %). This result indicates that the tea pigments extracted using ChCl-UA are purer. Compared with the Py**-**GC–MS profiles of the water extract, the lines were smoother, and the characteristic peaks were not clearly observed for the NADES extract (Fig. 6G). Experimental results suggested that HPTPs extracted with ChCl-UA have better thermal stability and require to be cleaved at a higher temperature for detection. Moreover, the dialysis membrane facilitated the removal of some small molecules along with ChCl-UA, and the final HPTP sample was purer as a result.

#### 3.7.3 Thermal analysis of the HPTPs

**Fig. 7:**
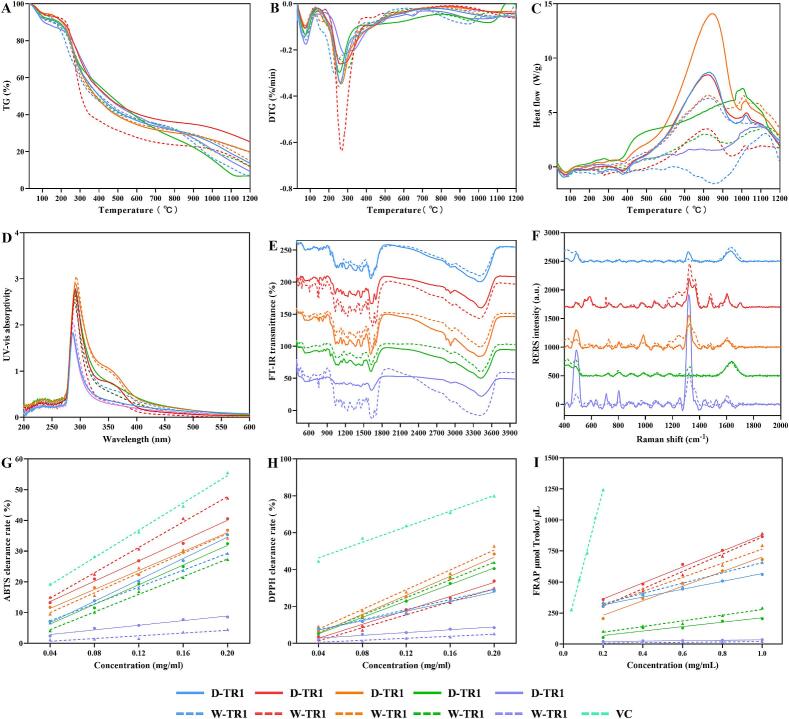
Thermal properties of tea pigments: TG (A), DTG (B), and DSC (C) curves. UV-visable (D), FT-IR (E), and SERS (F) data of tea pigments. Antioxidant activity results of the black tea pigments, as analyzed using ABTS (G), DPPH (H), and FRAP (I) assays.

TG, DTG, and DSC analyses (Fig. 7A-C) suggested that the quality of the tea pigments decreased with increasing temperature. However, differences were noted in the rate of quality reduction at different stages, as well as the heat absorption and release rates of the tea pigments. The thermally induced changes in the tea pigments can be broadly divided into three stages: first, during the rapid dehydration stage of 30–130 °C, the quality of the extracted tea pigments decreased with increasing temperature (5.48–11.04 %), as seen in the TG and DTG curves in Fig. 7A-C. Water evaporation results in an endothermic state, and the DSC data reveal that the tea-pigment extracts were all in an endothermic state at this stage (30–130 °C) (Xiao et al., 2020). Therefore, these mass reductions are possible due the evaporation of free and bound water in tea pigments, and the mass reduction rate reached the maximum at 64–78.5 °C. The second stage of mass loss at 130–400 °C involves the volatilization of organic matter, during which the changes in the materials are relatively complex. TG and DTG curves indicate that this process involves a rapid reduction in mass followed by a gradual one, and significant quality loss occurs in this stage (35.14–57.45 %). Tea pigments decompose to form abundant organic debris, which is lost at a maximum rate at 229–286 °C. DSC data indicated that the heat changes during this stage fluctuated owing to complex chemical and physical changes in both the endothermic and exothermic processes. Finally, the tea pigments continued to decompose further, and the decomposition products evaporated between 400 and 1200 °C. At this stage, the quality of the tea pigment decreases progressively. With the exception of W-TC, all tea pigments underwent exothermic reactions, and the ChCl-UA extracted tea pigment underwent greater exothermic reactions than the water-extracted tea pigment. Exothermic peak at 800 and 1000 °C for both samples, indicating that the high-temperature decomposition products of the tea pigments are similar, regardless of the extraction method. It is speculated that this final stage of decomposition includes exothermic reactions caused by the carbonization of organic matter and the decomposition of salts, such as carbonates. In summary, the peak temperature and intensity of the thermal degradation of the tea pigments obtained using the two extraction methods differed, but the thermal trends were similar.

### 3.8 Structural analysis of the HPTPs

#### 3.8.1 XRD and NMR spectroscopy

Crystalline materials exhibit sharp diffraction peaks, whereas amorphous ones generally exhibit a broad diffraction feature owing to the disordered arrangement of atoms. Both D-TP and W-TP showed broad XRD peaks (Fig. 6H-J), indicating that both were composed of amorphous polymers, and the higher the degree of disorder of amorphous polymers, the larger the width of the peaks (Xiao et al., 2023). Furthermore, the structural differences between the tea pigments in D-TP and W-TP were analyzed based on their chemical shifts in the ^1^H and ^13^C NMR spectra. The ^1^H NMR spectrum of tea pigment D-TP showed prominent chemical shifts at δ_H_ 3.05, 3.37, and 3.94. In contrast, tea pigment W-TP showed a greater number of peaks at δ_H_ 1.00, 3.08, 3.09, 3.10, 3.25, 3.50, 3.52, 3.54, 3.65, 3.91, 5.08, 6.87, and 7.53. The δ_H_ of the deuterated solvent, D_2_O, appeared at 4.70. W-TP showed more hydrogen chemical shifts, but D-TP showed lesser hydrogen chemical shifts with larger peak areas. D-TP showed a significant δ_H_ shift in the range of 3.00 to 4.00, indicating that it contains purer HPTPs. The removal of small- molecular substances during membrane separation decreased the number of ^1^H NMR peaks for D-TP. Some scholars have suggested that the chemical shifts of black tea extracts in the 1.00–2.00 and 6.50–7.50 ppm ranges correspond of those of amino acids, catechins, and other substances (Long et al., 2024). In addition, HPTP molecules have a basic skeleton of flavan-3-alcohol with high and uneven molecular weights, which results in extensive peak overlap in the NMR spectra. In brief, the NMR spectrum of tea pigment D-TP was more defined, and the significant chemical shifts of the carbon atoms of the tea pigment D-TP in ^13^C NMR were also more defined.

#### 3.8.2 UV–visible spectroscopy

In general, the UV–visible spectra of tea pigments show bands in the near-UV and visible regions. The characteristic UV–visible peaks of the tea pigments extracted using ChCl-UA and water were similar (Fig. 7D). In the near-UV region (200–400 nm), all 10 tea pigments had a prominent absorption peak at approximately 290 nm (Li et al., 2023). Both TR1 and TR2 exhibited shoulder peaks at 360 nm, which are typical and unique for TRs. However, at 360 nm, the absorbance of D-TR1 was significantly stronger than that of W-TR1. In addition, the absorption of TR2 in the near-UV region was significantly stronger than that in the other regions. This band may be attributed to UV absorption by the benzene ring or unsaturated bonds of the tea pigments, resulting in electronic transitions. In the visible range (400–600 nm), the absorbance of the tea pigments decreased with increasing wavelength. At wavelengths exceeding 400 nm, the absorbance of TRs decreased faster than that of TBs, and the absorbance of TB1 gradually increased compared with that of TB2. Therefore, TBs have a more notable effect on the solution color observed with naked eyes.

#### 3.8.3 FT-IR spectroscopy

The infrared spectra of the ChCl-UA- and water-extracted tea pigments were also similar (Fig. 7E). In addition, the characteristic peaks of TR1 and TR2 were evident, followed by the relatively broad characteristic peak of TB1, but the FT-IR spectral differences between D-TB2 and W-TB2 were significant. The tea pigments presented several common peaks, and 13 of them were selected for comparative analyses. FT-IR spectra are generally divided into fingerprint and energy cluster regions, and the fingerprint region includes benzene ring vibrations at 700–900 cm^−1^, along with other peaks located at 763, 825, and 874 cm^−1^. Ortho-, inter-, and para-disubstitutions of the external C—H moiety of benzene rings can affect the bending vibration of the benzene ring, leading to the appearance of characteristic peaks in the fingerprint region. The fingerprint area also includes the 1000–1475 cm^−1^ range, where in-plane C—H bending vibrations and stretching vibrations of single bonds such as C—O and C—C generally appear. The tea pigments exhibited characteristic peaks at 1035, 1146, 1232, 1364, and 1441 cm^−1^ in this region. The fingerprint region contained several absorption peaks that were difficult to assign; however, a large number of absorption peaks can reflect the characteristics of the extracted tea pigments.

The functional cluster region includes the double-bond stretching vibration region (1690–1500 cm^−1^), where a characteristic peak was observed at 1629 cm^−1^, possibly due to the stretching vibrations of C=C bonds and the skeletal vibrations of the benzene ring (Peng et al., 2013). The carbonyl stretching region (1900–1650 cm^−1^) contained an absorption peak at 1698 cm^−1^, indicating the presence of aromatic aldehydes (R-Ar-CO-H), which are carbonyl-containing aromatic compounds formed by polyphenolic polymers, such as catechins. The peak at 2854 cm^−1^ is related to the C—H bond. Further, the strong and broad peak at 3400 cm^−1^ is the characteristic absorption peak of O—H stretching vibrations that indicates that tea pigments are polyhydroxy polymers (Xiao et al., 2020).

#### 3.8.4 SERS

Raman spectroscopy is suitable for detecting nonpolar bonds, with each peak corresponding to a specific molecular bond vibration. Using the characteristic Raman peaks, a chemical fingerprint of a specific molecule can be formed. A comparative analysis of the multiple characteristic peaks in the Raman spectra of the HPTPs was performed (Fig. 7F). The spectra included the molecular vibrations of aromatic C=C bonds at 1610 cm^−1^, C—H bonds at 1324 cm^−1^, quinones at 1170 cm^−1^, and cyclic hydrocarbons at 1460–1150 cm^−1^. HPTPs are substances composed of C—H bonds, carbonyl groups (C=O), and unsaturated benzene ring structures. The Raman results indicate that the characteristics of the tea pigments obtained using the two extraction methods are similar. The characteristic peaks of TR1 and TR2 were clearly noticeable, whereas the peaks of TP and TB1 were relatively broad. In addition, a significant difference was observed in the peak intensities of D-TB2 and W-TB2. This result is similar to the FT-IR result.

### 3.9 Antioxidant properties of HPTPs

Tea pigments are not only important substances that affect the color of the tea brew, but they also have a certain nutritional value. The results in Fig. 7G-I indicate that the tea pigments extracted using ChCl-UA have excellent antioxidant properties. However, the antioxidant capacity of vitamin C (V_C_) was better than that of the tea pigments. The analysis revealed that TR1 has the best antioxidant capacity in the ABTS and FRAP assays, followed by TR2. TRs are formed by the polymerization of substances such as catechins and TFs, which have good antioxidant capacity; thus, TRs have good antioxidant activity. In addition, the TB1 component is more complex, and its antioxidant capacity was lower in the ABTS and FRAP assays, which may be due to the lower content of polyphenols and other antioxidants (Liu et al., 2022; Tang et al., 2019). However, TB1 was found to have a high antioxidant capacity in the DPPH assay. The antioxidant capacity of tea pigment TP was affected by TB2 (low antioxidant capacity) and was lower than those of TR1 and TR2. In conclusion, extraction with ChCl-UA can better retain the active components of the tea pigments.

## 4 Conclusion

The effectiveness of a NADES in the extraction of HPTPs from black tea was demonstrated. Considering the structural complexity of tea pigments, their extraction using ChCl-UA as a selected NADES was compared with water extraction to verify the effectiveness of NADES extraction. The results revealed that the extraction of tea pigments using ChCl-UA is more efficient than the traditional water extraction, and the separation of the ChCl-UA from HPTPs could be effectively realized using a membrane with an appropriate molecular weight cut-off. In terms of spectral characteristics, the extracted samples were found have similar functional groups, but the characteristic intensities were different. In terms of the microscopic morphology and color characterization of the extracted samples, the structures of the pigments were similar; however, the tea pigments obtained using ChCl-UA had a higher degree of polymerization. Compared with the sample extracted using water, the HPTPs sample extracted using the NADES was similar in composition and thermal properties. However, the HPTPs obtained using the ChCl-UA were more abundant. Finally, the analysis of the antioxidant activity revealed that the HPTPs obtained using ChCl-UA had effective antioxidant properties. The characterization and analysis of the pigments in black tea deepen our understanding of TRs and TBs, and partially reveal the effect of ChCl-UA on HPTPs extraction. Further research is required to elucidate the effects of ChCl-UA on small molecules during membrane separation. The structural information of the key substances in the TRs and TBs extracted using ChCl-UA would advance the application of HPTPs in food, medicine, and cosmetics.

## CRediT authorship contribution statement

**Di Wang:** Writing – original draft, Validation, Methodology, Data curation. **Yang Liu:** Writing – review & editing, Formal analysis, Conceptualization. **Bin Zeng:** Funding acquisition, Formal analysis. **Yuqin Xu:** Methodology, Investigation. **Sheng Cao:** Validation, Data curation. **Yuanyan Luo:** Project administration, Methodology. **Shuangling Xiao:** Validation, Methodology. **Jie Teng:** Writing – review & editing, Funding acquisition, Conceptualization.

## Declaration of competing interest

The authors declare that they have no known competing financial interests or personal relationships that could have appeared to influence the work reported in this paper.

## Data Availability

Data will be made available on request.

## Appendix A Supplementary data

Supplementary material Image 1
