# Supplementary material for: Compositional and biochemical activity evaluation of highly polymerized tea pigments in black tea based on natural deep eutectic solvent extraction
Source: Food Chem X. 2025 Mar 25;27:102413. doi: 10.1016/j.fochx.2025.102413 (PMC11999212; doi:10.1016/j.fochx.2025.102413)
Supplement: Supplementary file 1 — Supplementary material [file mmc1.docx]

**

**

**Fig. S1.** Fourier transform infrared spectrum (FT-IR) of NADESs.





**Fig. S2.** Surface-enhanced Raman scattering (SERS) results of the NADESs.

**
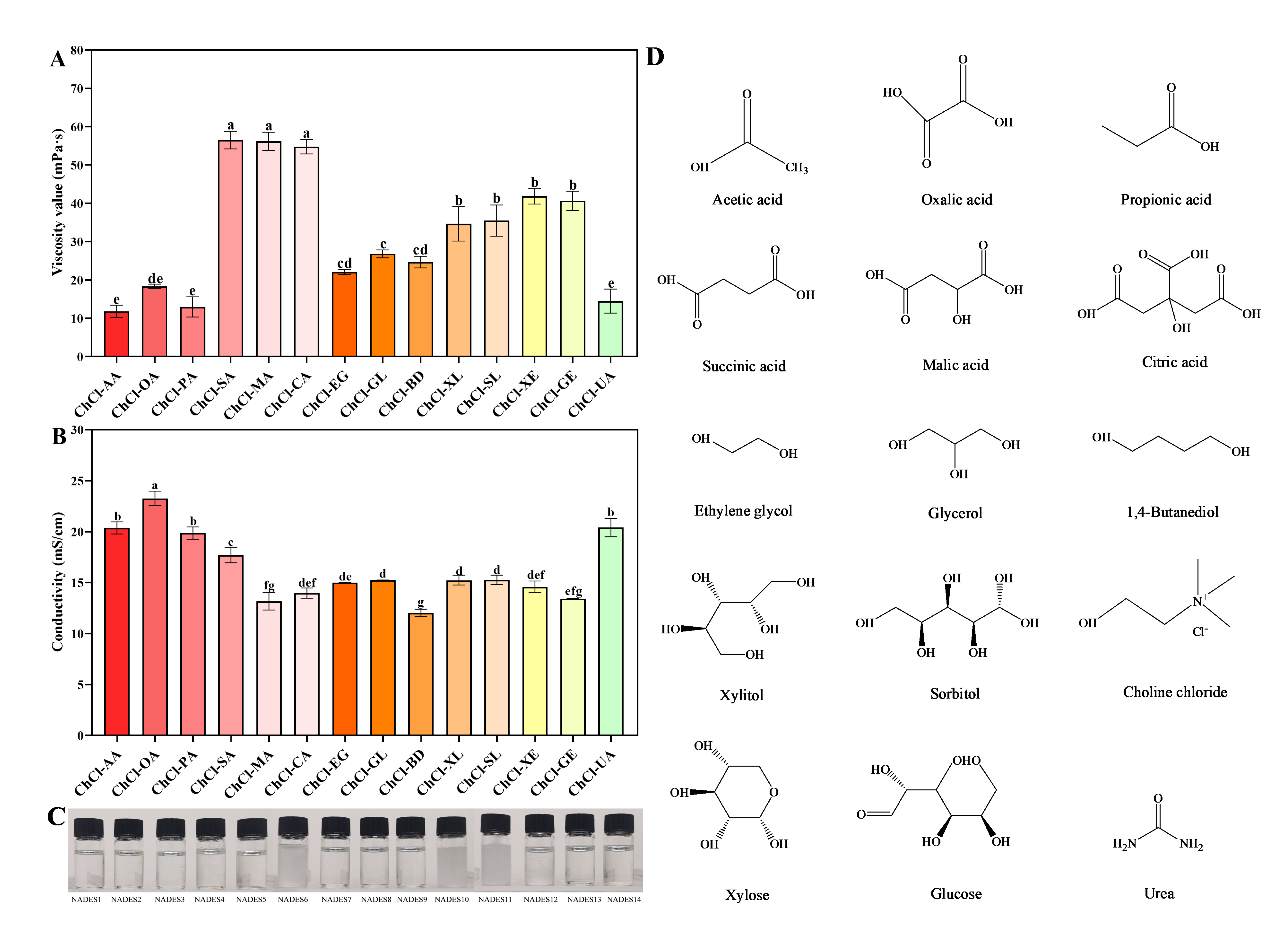
**

**Fig. S3.** Viscosity (A), conductivity (B), and physical diagram (C) of the NADESs. Chemical structures of the hydrogen-bonding acceptor (HBA) and hydrogen-bonding donors (HBDs) (D).

**Table S1**

Establishment and analysis of the response surface model for the extraction of tea pigments using choline chloride-urea (ChCl-UA) as the NADES.

| Level | A: NADES water content  (%) | B: Extraction time (min) | C: Extraction temperature (℃) |
| --- | --- | --- | --- |
| –1 | 20 | 13 | 70 |
| 0 | 30 | 16 | 80 |
| 1 | 40 | 19 | 90 |

**Table S2**

Response surface analysis test and results.

| No. | A: NADES water content (%) | B: Extraction time (min) | C: Extraction temperature (℃) | Y: Extraction rate (%) |
| --- | --- | --- | --- | --- |
| 1 | 0 | 0 | 0 | 41.24 |
| 2 | 0 | 0 | 0 | 41.14 |
| 3 | 0 | 0 | 0 | 41.38 |
| 4 | 0 | –1 | 1 | 37.76 |
| 5 | –1 | 1 | 0 | 35.83 |
| 6 | 1 | 0 | –1 | 34.69 |
| 7 | 0 | 0 | 0 | 40.90 |
| 8 | 1 | 0 | 1 | 39.44 |
| 9 | 0 | 0 | 0 | 41.23 |
| 10 | –1 | 0 | 1 | 36.47 |
| 11 | 1 | –1 | 0 | 34.94 |
| 12 | –1 | 0 | –1 | 36.28 |
| 13 | 1 | 1 | 0 | 39.41 |
| 14 | 0 | 1 | –1 | 36.06 |
| 15 | 0 | –1 | –1 | 35.40 |
| 16 | 0 | 1 | 1 | 39.17 |
| 17 | –1 | –1 | 0 | 38.13 |

**Table S3**

Variance analysis of regression model of the tea-pigment extraction rate.

| Source of variance | Sum of squares | Freedom | Mean square | F value | P value | Significance |
| --- | --- | --- | --- | --- | --- | --- |
| model | 95.94 | 9 | 10.66 | 266.41 | <0.0001 | ** |
| A– NADES water content | 0.39 | 1 | 0.39 | 9.79 | 0.0166 | * |
| B– Extraction time | 2.27 | 1 | 2.27 | 56.72 | 0.0001 | ** |
| C– Extraction Temperature | 13.54 | 1 | 13.54 | 338.41 | <0.0001 | ** |
| AB | 11.46 | 1 | 11.46 | 286.42 | <0.0001 | ** |
| AC | 5.20 | 1 | 5.20 | 129.95 | <0.0001 | ** |
| BC | 0.14 | 1 | 0.14 | 3.52 | 0.1028 |  |
| A^2^ | 21.06 | 1 | 21.06 | 526.39 | <0.0001 | ** |
| B^2^ | 14.59 | 1 | 14.59 | 364.65 | <0.0001 | ** |
| C^2^ | 20.71 | 1 | 20.71 | 517.66 | <0.0001 | ** |
| Residual | 0.28 | 7 | 0.04 |  |  |  |
| Omission item | 0.15 | 3 | 0.05 | 1.65 | 0.3135 |  |
| Pure error | 0.13 | 4 | 0.03 |  |  |  |
| Summation | 96.22 | 16 |  |  |  |  |
| Standard deviation =0.20; C.V. %=0.52; Signal to Noise Ratio =35.11; | | | | | | |
| R^2^=0.9971; R^2^_adj_=0.9933; R^2^_pred_=0.9722 | | | | | | |

**Table S4.** Color difference analysis of the tea pigments.

| Name | Image | L* | a* | b* | C* | H* |
| --- | --- | --- | --- | --- | --- | --- |
| D-TC | **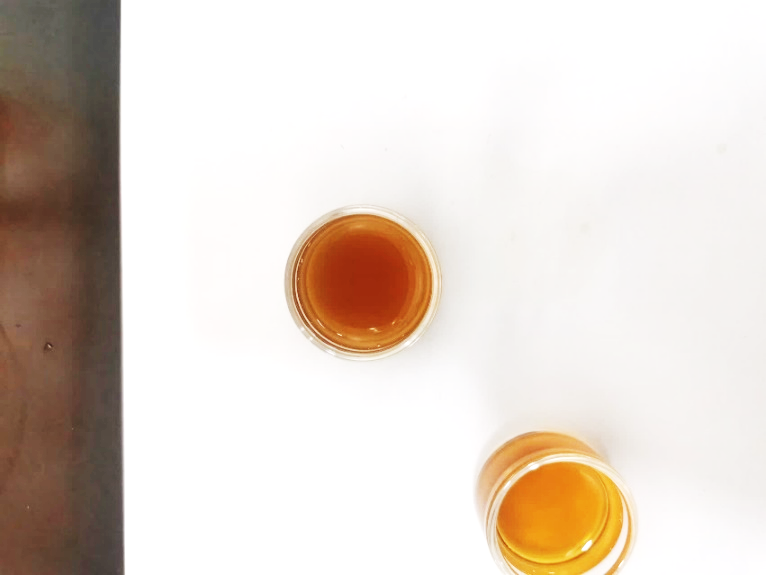** | 23.28±  0.04^i^ | 8.53±  0.1^b^ | 12.77±  0.1^h^ | 15.35±  0.14^h^ | 56.27±  0.19^j^ |
| D-TR1 | **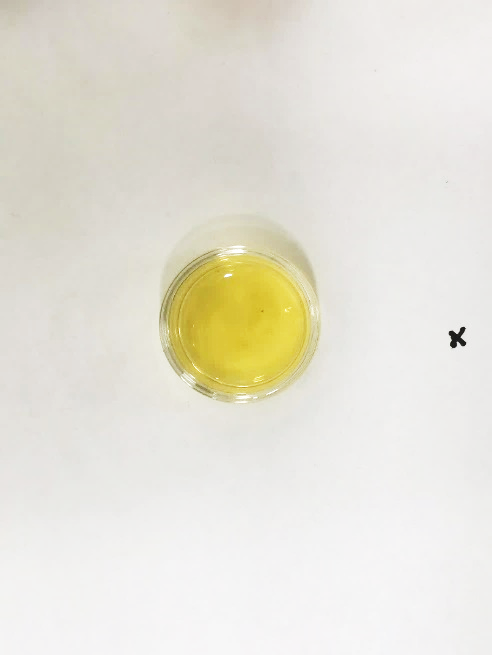** | 62.07±  0.03^b^ | -2.25  ±0.02^f^ | 30.04  ±0.02^d^ | 30.13  ±0.02^d^ | 94.29  ±0.04^b^ |
| D-TR2 | **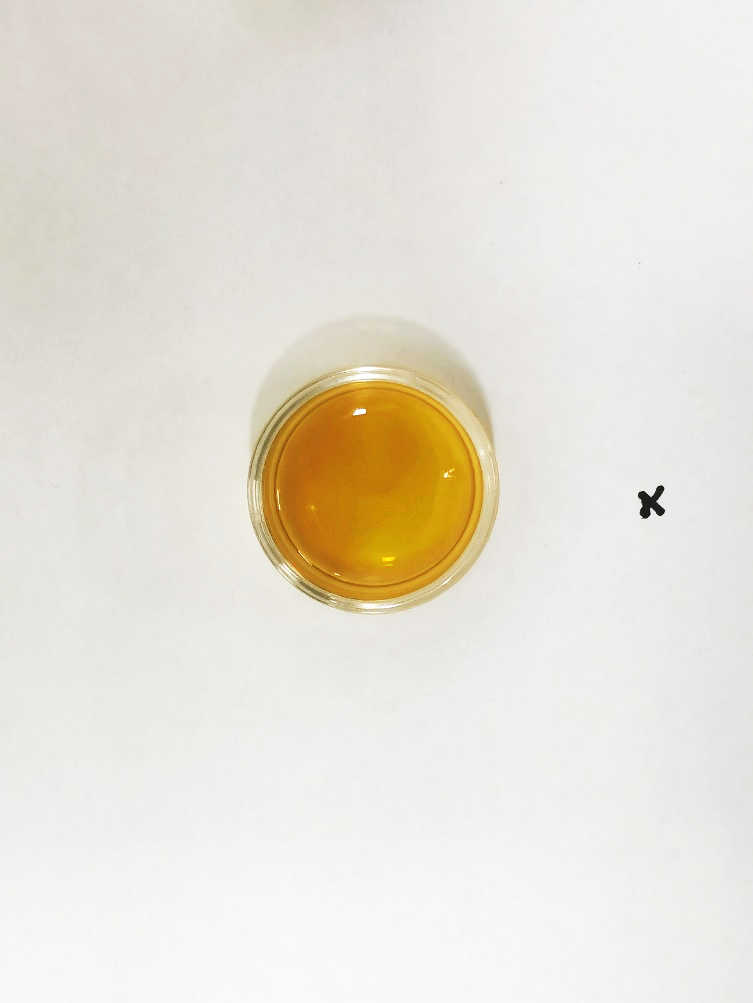** | 45.83  ±0.09^e^ | 8.71  ±0.02^b^ | 39.39  ±0.14^b^ | 40.34  ±0.13^b^ | 77.54  ±0.07^e^ |
| D-TB1 | **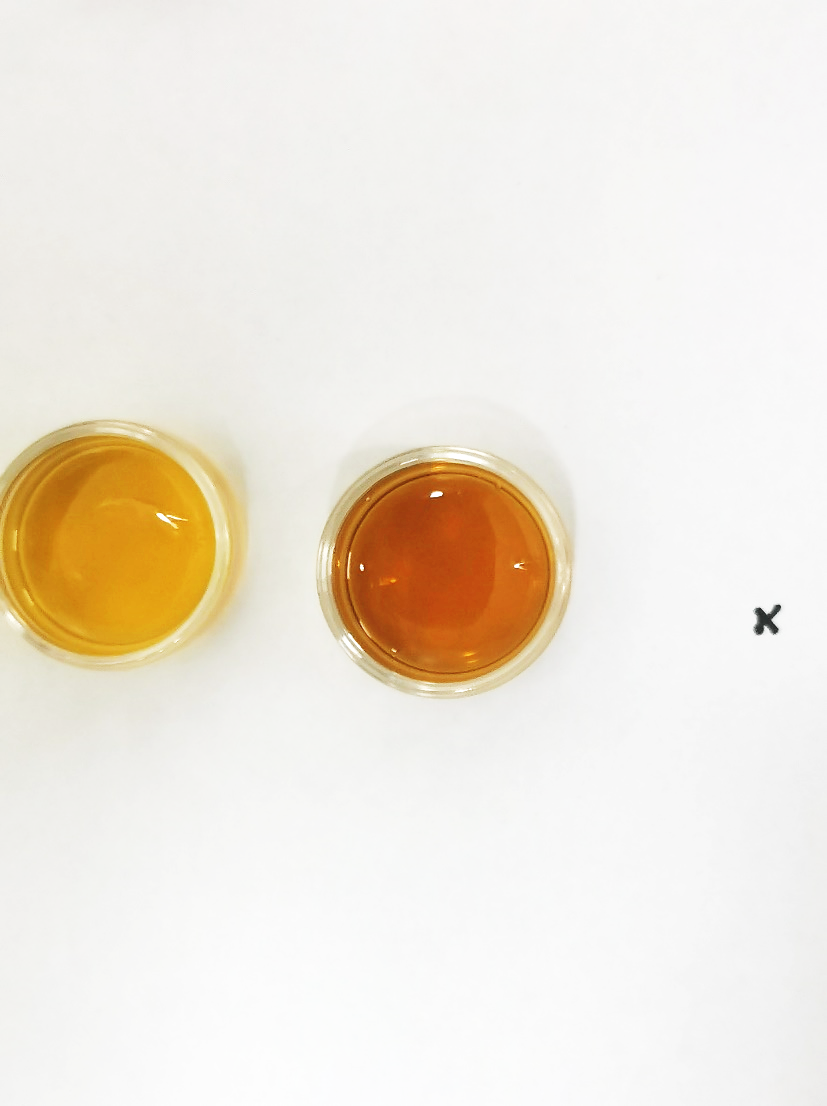** | 32.21  ±0.26^g^ | 11.66  ±0.1^a^ | 25.68  ±0.25^e^ | 28.2  ±0.27^e^ | 65.58  ±0.09^i^ |
| D-TB2 | **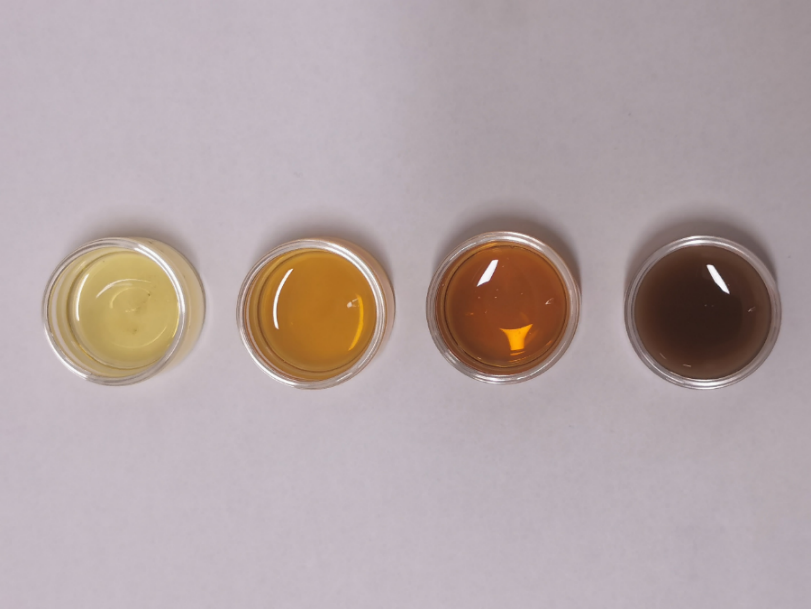** | 20.33  ±0.05^j^ | 2.45  ±0.06^e^ | 6.69  ±0.05^i^ | 7.12  ±0.04^j^ | 69.92  ±0.58^h^ |
| W-TC | **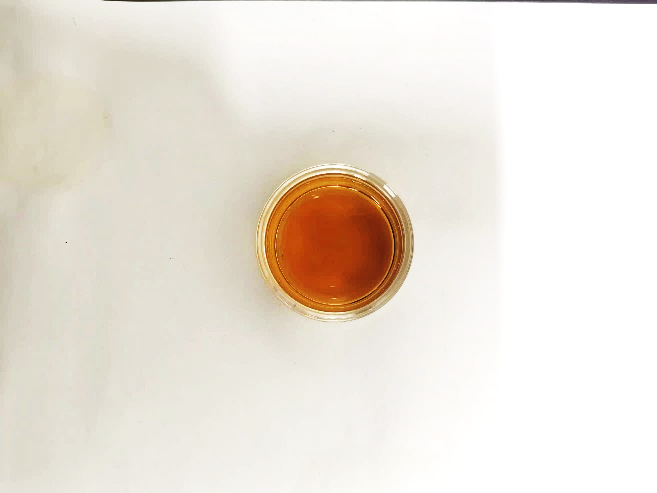** | 37.45±  0.4^f^ | 11.32±  0.17^a^ | 32.22±  0.33^c^ | 34.15±  0.37^c^ | 70.65±  0.09^g^ |
| W-TR1 | **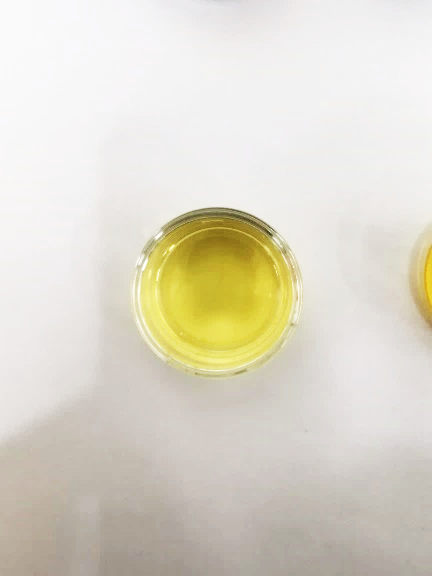** | 69.57  ±0.54^a^ | -3.43  ±0.11^g^ | 24.86  ±0.12^f^ | 25.09  ±0.12^f^ | 97.85  ±0.23^a^ |
| W-TR2 | **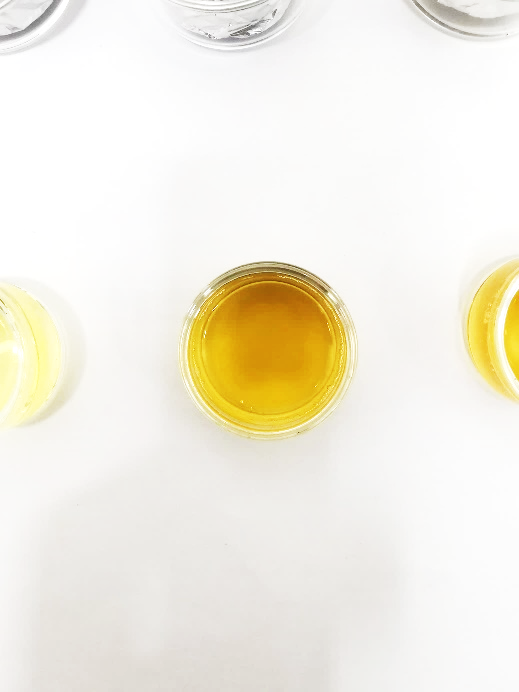** | 56.49  ±0.45^c^ | 3.01  ±0.07^d^ | 43.44  ±0.28^a^ | 43.55  ±0.28^a^ | 86.04  ±0.11^c^ |
| W-TB1 | **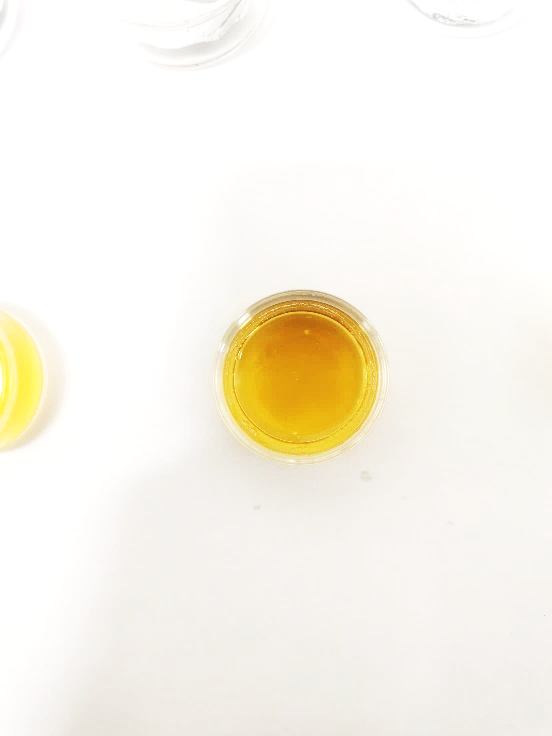** | 53.09  ±0.4^d^ | 5.4  ±0.13^c^ | 39.43  ±0.28^b^ | 39.8  ±0.29^b^ | 82.2  ±0.14^d^ |
| W-TB2 | **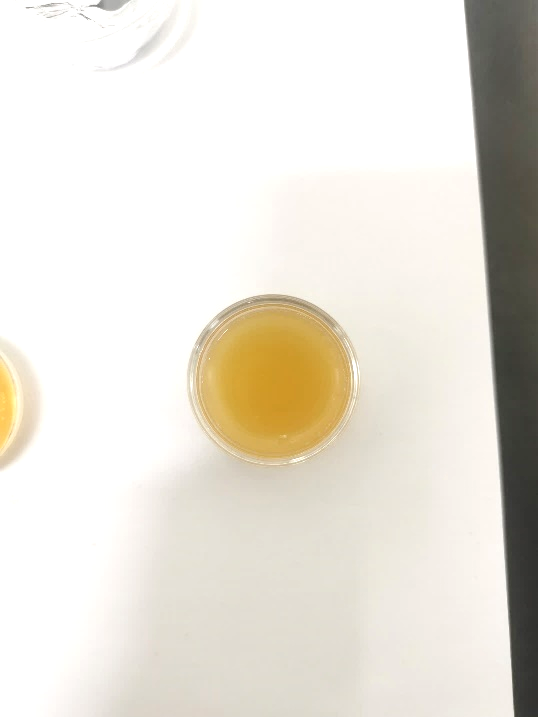** | 27.23  ±0.27^h^ | 5.58  ±0.1^c^ | 17.38  ±0.39^g^ | 18.26  ±0.39^g^ | 72.2  ±0.24^f^ |

**Table S5**

Analysis of the Py-GC-MS data of black tea pigments (D-TP).

| No. | Rt (min) | Compound ID | Area (%) |
| --- | --- | --- | --- |
| 1 | 0.241 | Methane | 0.048 |
| 2 | 0.347 | Ammonia | 0.278 |
| 3 | 0.911 | Water | 0.194 |
| 4 | 1.475 | Ketene | 41.479 |
| 5 | 3.978 | 1,6-Diaminohexane-N,N,N',N'-tetraacetic acid | 9.130 |
| 6 | 25.003 | Caffeine | 48.871 |

**Table S6**

Analysis of the Py-GC-MS data of black tea pigments (W-TP).

| No. | Rt (min) | Compound ID | Area (%) |
| --- | --- | --- | --- |
| 1 | 3.954 | Carbon dioxide | 6.421 |
| 2 | 7.348 | 2-Propanamine | 0.036 |
| 3 | 8.018 | Urea | 0.012 |
| 4 | 8.591 | 1-Methyl-2-phenoxyethylamine | 0.009 |
| 5 | 12.311 | 2-Amino-1-(o-hydroxyphenyl)propane | 0.007 |
| 6 | 12.39 | Benzenepropanamine, α-methyl- | 0.007 |
| 7 | 12.487 | 3-Methoxyamphetamine | 0.009 |
| 8 | 12.681 | Phenethylamine, p,.alpha.-dimethyl- | 0.012 |
| 9 | 12.795 | Acetic acid, hydroxy[(1-oxo-2-propenyl)amino]- | 0.015 |
| 10 | 12.928 | 2-Amino-1-(o-hydroxyphenyl)propane | 0.018 |
| 11 | 13.368 | Phenol | 0.287 |
| 12 | 13.589 | Phenol | 0.189 |
| 13 | 14.232 | Phenol | 0.030 |
| 14 | 14.373 | Phenol | 0.010 |
| 15 | 14.426 | Phenol | 0.009 |
| 16 | 14.479 | Phenol | 0.009 |
| 17 | 14.541 | Phenol | 0.012 |
| 18 | 14.638 | Phenol | 0.024 |
| 19 | 14.7 | Acetic acid, phenyl ester | 0.019 |
| 20 | 14.744 | Carbamic acid, methyl-, phenyl ester | 0.010 |
| 21 | 14.77 | Acetic acid, phenyl ester | 0.013 |
| 22 | 14.841 | Phenol | 0.017 |
| 23 | 15.123 | Metaraminol | 0.079 |
| 24 | 15.167 | Metaraminol | 0.017 |
| 25 | 15.255 | 2,3-Dimethoxyamphetamine | 0.035 |
| 26 | 15.387 | Benzeneethanamine, 4-methoxy-.alpha.-methyl- | 0.080 |
| 27 | 15.511 | 2,4-Dimethylamphetamine | 0.113 |
| 28 | 15.934 | N-[3,5-Dinitropyridin-2-yl]proline | 0.022 |
| 29 | 16.066 | Metaraminol | 0.023 |
| 30 | 16.233 | Metaraminol | 0.007 |
| 31 | 16.33 | 1,2,3,4-Butanetetrol, [S-(R*,R*)]- | 0.008 |
| 32 | 17.115 | 2-Amino-1-(o-methoxyphenyl)propane | 0.011 |
| 33 | 18.596 | 2-Amino-5H-pyrrolo[3,4-d]pyrimidine-4,7(3H,7H)-dione | 0.090 |
| 34 | 19.09 | Benzene, [(2-methylpropyl)thio]- | 0.247 |
| 35 | 20.553 | Hydroquinone | 0.102 |
| 36 | 20.703 | Resorcinol | 0.385 |
| 37 | 21.479 | Hydroquinone | 0.008 |
| 38 | 22.475 | 4-Acetylphenyl ether | 8.280 |
| 39 | 22.73 | 9H-Thioxanthen-9-one, 2-(1-methylethyl)- | 3.984 |
| 40 | 23.074 | Benzo(a)pyrene, 7,8-dihydro- | 1.243 |
| 41 | 23.136 | 9H-Thioxanthen-9-one, 2-(1-methylethyl)- | 2.313 |
| 42 | 23.515 | Methanone [4-(1,1-dimethylethyl)phenyl](4-hydroxyphenyl)- | 1.778 |
| 43 | 23.806 | 4-Acetylphenyl ether | 1.441 |
| 44 | 24.097 | 9,10-Anthracenedione, 1-amino-4-hydroxy- | 0.814 |
| 45 | 24.449 | Benzaldehyde, 3-[4-(1,1-dimethylethyl)phenox]- | 0.565 |
| 46 | 24.652 | Hydroquinone bis(trimethylsilyl) ether | 0.213 |
| 47 | 24.969 | Caffeine | 41.508 |
| 48 | 30.32 | 9H-Thioxanthen-9-one, 2-(1-methylethyl)- | 8.839 |
| 49 | 31.316 | Hexadeca-2,6,10,14-tetraen-1-ol, 3,7,11,16-tetramethyl- | 12.661 |
| 50 | 33.071 | 2-Azetidinone, 1-(diphenylacetyl)-3,3,4-triphenyl- | 7.958 |
